# Recent advances in minimally invasive biomarkers of OSCC: from generalized to personalized approach

**DOI:** 10.3389/froh.2024.1426507

**Published:** 2024-08-02

**Authors:** Smriti Suri, Geeta S. Boora, Rajandeep Kaur, Anshika Chauhan, Sushmita Ghoshal, Arnab Pal

**Affiliations:** ^1^ Department of Biochemistry, Postgraduate Institute of Medical Education and Research, Chandigarh, India; ^2^ Department of Radiotherapy, Postgraduate Institute of Medical Education and Research, Chandigarh, India

**Keywords:** oral squamous cell carcinoma (OSCC), minimally invasive biomarker, proteomic biomarker, genomic biomarkers, circulating tumour cell (CTC), circulating tumour DNA (ctDNA)

## Abstract

Oral cancer is the 6th most common type of cancer worldwide, and oral squamous cell carcinoma (OSCC) accounts for >90% of oral cancers. It is a major health problem, particularly in low- and middle-income countries (LMICs), due to both its high incidence and significant mortality and morbidity. Despite being a global burden, and even with the significant advancement in the management of OSCC, the overall outcome of the disease is still abysmal. With the advent of time, advanced diagnostic and treatment approaches have come into practice, but the burden of the disease has not improved significantly. Major reasons attributed to the poor outcome are delay in diagnosis, locoregional recurrence and resistance to the currently available treatment regimen. In this review, we have highlighted the existing challenges in the diagnosis and have emphasized the advancements in minimally invasive biomarkers. Additionally, the importance of collaborative multidimensional approaches involving clinicians and researchers has been discussed, as well as the need to redefine and establish better utility and management of existing diagnostic and treatment protocols along with the minimally invasive/non-invasive biomarkers.

## 1 Introduction

Oral squamous cell carcinoma (OSCC) is a global health problem and is the sixth most common cancer worldwide, with a global incidence of 389,846 cases in the year 2022. Among those cases, the majority number of cases (258,440) were observed in the Asian subcontinent (1). According to Globocan 2022 estimates, the disease accounts for 188,438 deaths, approximately 75% of them are from Asia (1). With a highly disproportionate burden in low-and middle-income countries (LMICs), it accounts for >25% of all cancer in certain regions globally (2). OSCC is characterized by neoplastic transformation of the epithelium of the oral cavity. The lesions are associated with pain, ulceration, nodularity, and irregular floor and margins, which are often hard upon palpitation (3, 4). Although the disease has a multifactorial etiology, it is majorly caused by smoking, betel nut chewing, alcohol consumption, nutritional deficiencies, poor oral hygiene, traumatic injury of dental origin and viral infections with HPVs and has a significant male preponderance (4). Surprisingly, the combination of alcohol and smoking abuse has led to 11 million deaths annually, leading to a synergistic increase in cases (5).

## 2 Traditional approaches towards the management of OSCC

The majority of the cases are diagnosed primarily by clinical examination and confirmed with tissue biopsy of the lesion and prognosticated by TNM staging after radiological and histological examination. The current treatment modalities for OSCC are surgery with or without removal of lymph nodes by neck dissection, followed by radiotherapy and/or chemotherapy. In cases of non-metastatic and early-stage disease, surgery remains the primary treatment approach. In contrast, patients with a later disease stage and with high risk of recurrence are treated with radiation therapy (RT) and/or chemoradiotherapy (CRT) in addition to surgery as an adjuvant approach (6). At times, patients who are unfit for surgery are given RT/CRT as the primary treatment modality (7). Systemic therapy involves usage of chemotherapeutic agents like docetaxel and cisplatin where surgery cannot be performed or in higher-stage cancers (8). Surgery, radiotherapy, and chemotherapy are the first line of treatment methods for OSCC, but since last few years, immunotherapy has also come in power. In 2016, the U.S. food and drug administration (FDA) approved two PD-1 immune checkpoint inhibitors nivolumab (Opdivo) and pembrolizumab (Keytruda) to be used in the treatment of relapsed and refractory HNSCC, also few more antibodies against PD-1 have been approved for marketing in the US (9). Compared to other cancers, the 5-year survival rate of OSCC is poor, ranging approximately from 20% to 80% based on the stage at which the disease is diagnosed (10, 11). However, the majority of the cases are diagnosed at a later stage, leading to an overall inferior outcome.

## 3 Challenges associated with currently available management approaches

Various technological advancements have been made in the field of diagnosis to facilitate timely screening, staging, and monitoring of the disease and initiate therapeutic response, in last two decades. The diagnostic approaches for OSCC can be categorized in two major groups viz tumour imaging techniques and molecular diagnostic techniques (12). X-rays, computed tomography (CT), positron emission tomography (PET), single photon emission computed tomography (SPECT), and magnetic resonance imaging/spectroscopy (MRI/MRS) are among the tumour imaging techniques used for diagnosis of OSCC. In contrast, at the molecular levels, techniques such as next generation sequencing (NGS), assessment of genomic alteration via liquid biopsy using circulating tumour cells (CTCs), circular tumour DNA (ctDNA), non-coding RNAs, exosomes and tissue-based approach via immunohistochemistry (IHC) and spatial transcriptomics are used. Tumour imaging techniques majorly deal with the anatomical aspects including size, shape involvement of nearby tissues etc., the molecular diagnostics report the functional aspect of the tumour in cellular and subcellular levels by analyzing metabolomic, proteomic, genomic and transcriptomic signatures of cancer cells (13–16). However, most of the molecular diagnostic aspects of OSCC are currently confined to laboratory setup (17).

Despite several management approaches, timely diagnosis and treatment of OSCC still remains a challenge. It has been noticed that a significant number of patients are unaware of the condition, and the disease remains undetected till it grows in size and stage and becomes visibly identifiable; this delay might be due to a lack of awareness or seriousness among patient groups towards the initial symptoms (18). Also, at times, there is a sense of denial among patients that leads to delays in seeking medical advice at the incipient stage of the disease, further worsening the condition (19). Furthermore, patients with higher tumour stage (T3–T4), advanced histological grading (moderate or poor differentiation), lymph node positivity (N2–N3) and progressive vascularization show an early relapse of the disease (20, 21). As in a good number of patients, the disease gets diagnosed at later stages, its curative management becomes challenging, and patients often present with incomplete remission or early recurrence.

## 4 Scope of improvement in diagnostics-emerging role of minimally invasive biomarkers

For a normal healthy cell to become malignant, various alterations in its genome, epigenome, and transcriptome are involved. The pattern of these alterations may vary from patient to patient and will ultimately play an important role in disease progression as well as in the treatment response (22). Many times, the alterations are at different levels simultaneously involving genome, epigenome, transcriptome etc., making the tumour tissue with a heterogeneous population of cancer cells with different characteristics. Moreover, these characteristics are also dynamic in nature and change in temporo-spatial fashions. These heterogeneous characteristics of tumours indicate the imperative need for personalized cancer management. The detection of the pattern in the form of changes in the level of biomarker in conjunction with the current diagnostic protocol is thus important for designing a personalized therapy to improve the disease outcome (23).

### 4.1 Biomarkers for OSCC

National Cancer Institute (NCI) states that a biomarker is “a biological molecule found in blood, other body fluid, or tissues that is a sign of a normal or abnormal process, or of a condition or disease” (24). Specifically, a cancer biomarker is an entity that quantifies as an indicator of risk and occurrence of cancer and helps predict patient outcomes to a modality (25). Within the body, there can be a varied range of molecules with altered levels and phenotypes indicative of different conditions. These molecules help to differentiate diseased subjects from non-diseased healthy individuals (26). After undergoing various steps in the process of discovery and development, from analytical validation and evidentiary assessment qualification to utilization, the molecule can serve as a biomarker and qualifies as the primary basis for regulatory approval for marketing (27).

With the advent of time and advancement in the field of tumour diagnostics, several tumour biomarker research experts published “REporting recommendations for Tumour MARKer prognostic studies” (REMARK) criteria, with an aim to provide guidelines while assessing a biomolecule to be considered as a biomarker and reporting of Tumour Biomarker Tests (TBT) associated studies. Along with that, later on, to enhance transparency on biospecimen collection, processing and archiving, Biospecimen Reporting for Improved Study Quality (BRISQ) criteria were developed (28).

A variety of samples can be used to study cancer biomarkers. Tumour tissue, the most invasive among others, is the most reliable and widely used sample type for biopsies so far. Since cancer is a heterogeneously evolving disease associated with metastasis, it is preferred to understand the spatial and temporal evolution by looking at multiple biopsies to get a holistic approach to the disease management in real time (29). However, obtaining resected tissue through invasive procedures at regular intervals for molecular profiling of the disease is associated with various challenges (30). It is not feasible to invasively acquire patient tumour tissue samples repeatedly throughout the course of treatment to monitor response and relapse (29). Considering the various challenges and shortcomings associated with traditional biopsy approaches, current analysis of tumour progression is being complemented by less invasive and more convenient approaches of liquid biopsy (31). Liquid biopsy is a technique of analyzing various biological fluids such as blood, saliva, pleural fluids, urine and cerebrospinal fluids (CSF), to get a real-time picture of tumour status by focusing on different tumour-derived components such as circulating tumour cells (CTCs), circulating tumour DNA (ctDNA), and tumour extracellular vesicles (EVs) (32, 33). Since a great emphasis in current cancer diagnosis is on finding less invasive and cost-effective strategy for early diagnosis and improved prognosis, looking for biomarkers using liquid biopsy can provide a more comprehensive management of the disease.

Minimally invasive/non-invasive biomarker approaches are at the forefront of personalized cancer care, which involves customizing diagnostic or prognostic evaluations for the needs of each patient according to the specific features of the cancer and their associated clinical profile (34). Likewise, it has been found that the newer targeted therapy, including inhibitors of *EGFR, ALK, PI3K/AKT/mTOR, RAS-MAPK, RET, MET, BRAF,* and *NTRK/ROS1*, as well as *PD1* and *CTLA4* molecules, for non-small cell lung cancer (NSCLC) have far better treatment responses than the standard therapies (35). Similar strategies such as use of anti-EGFR agents like cetuximab in case of OSCC patients with CTC expressing EGFR may be adopted in the management of OSCC (36).

#### 4.1.1 Types of biomarkers in OSCC

##### 4.1.1.1 Based on the nature of the molecule

Different biological fluids such as blood, saliva, pleural fluid, urine, and cerebrospinal fluid (CSF) can be analyzed to study a wide array of tumour-derived moieties in the form of biomarkers for OSCC (33). For OSCC, saliva and serum are the most reliable and explored biological fluids for the study of biomarkers. Saliva is the closest contact fluid to the tumour in OSCC and contains an array of analytes such as cytokines, enzymes, and antibodies, which makes it an appropriate indicative body fluid for biomarker analysis and, hence, can be a useful alternative to serum and tissue testing (37). In addition, its accessibility, ease of collection and non-invasive nature make it one of the most extensively used fluids for the investigation of biomarkers in OSCC (38).

Serum, on the other hand, is also widely explored as it contains a pool of biomolecules that have been shed off from the site of the tumour into the circulation, which can be analyzed to get clinically valuable information on both the patient and their underlying malignancy (39). The biomarkers obtained from these biological fluids vary in nature depending upon the type of modifications, such as genomic, epigenomic, transcriptomic, proteomic and cellular.

###### 4.1.1.1.1 Genomic, epigenomic and transcriptomic biomarkers

As is widely known, cancer develops from different types of alterations in DNA, modifying their structure to a great extent. These modifications can be at small scale, such as DNA base insertions, substitution and deletion or large-scale rearrangements in DNA, such as gene duplications/deletions, chromosomal inversions/translocations and loss of heterozygosity (40). Hence, profiling the tumour can help understand the genomic alterations and epigenomic regulations that have influenced the growth and development of cancer.

Alterations in the genome due to a single base pair, such as a point mutation at tumour suppressor gene *p53* exon 4 codon 63, have been proven to be significantly associated with the pathophysiology of OSCC and ought to be a good diagnostic biomarker (41). Also, it was reported that the karyotypes of OSCC patients are composed of multiple numerical and structural abnormalities (42). In OSCC patients, heterogeneity in the saliva at the DNA microsatellite level has been correlated with their tumour specimen, and ctDNA isolated from the saliva has also been shown as an early diagnostic biomarker (43). In one of the studies by our group, genomic instability found in tumour DNA isolated from the saliva of OSCC patients was shown to act as a predictive biomarker for response to treatment by radiotherapy (44). Furthermore, DNA methylation also played a major role and can be used as a powerful diagnostic approach to scrutinize OSCC patients from healthy controls. Ferlazzo et al. and Demokan et al., individually in their respective studies, showed a higher frequency of *p16, MGMT* and *K1F1A,* and *EDNRB* promoter methylation in the saliva of their OSCC patients' groups than healthy controls (45, 46).

Methylation of *SHOX2* and *SEPT9* in cfDNA derived from serum samples of HNSCC patients has shown promising diagnostic potential (47). Mydlarz et al., in their study on OSCC patients, concluded that hypermethylation of cfDNA at *EDNRB* gene derived from serum has a role in the detection of OSCC patients with 100% specificity, however with low sensitivity (48). Various studies have also found that levels of cfDNA have a direct correlation with disease severity. Higher cfDNA in tumour samples from locally advanced HNSCC patients had proportionally gross tumour volume (49). Also, in another group of 121 OSCC patients, a higher plasma level of cfDNA was related to poor prognosis (50). In OSCC, several non-coding RNA family members, such as long non-coding (lnc)RNA and micro RNA (miRNA) and circular RNAs (circRNAs) from various body fluids, such as blood saliva, are found to be associated with disease severity. It was found in the literature that lncRNA such as *BLACAT1, AC104041.1, ADAMTS9-AS2, ANRIL, and BANCR,* isolated from cancerous cells were dysregulated and are involved in the promotion of growth, metastasis, proliferation, invasion, and migration (51–55). A study by Rajan et al. on miRNA expression profiling in OSCC among three external datasets in comparison to their data, published a list of various upregulated miRNA such as miR-196a, miR-196b, miR-155, miR-21, miR-424, and many downregulated miRNA such as miR-345, miR-101, miR-144, miR-204 (56). In OSCC, circRNA_100290 regulate the expression of *CDK6*, cause G1/S arrest and inhibit proliferation of the cells (57).

###### 4.1.1.1.2 Proteomic biomarkers

Proteins are the biomolecules translated from genes that regulate major cellular processes. Any change in the genes encoding these proteins, any modification in the splicing process or further post-translational modifications of the translated protein such as phosphorylation, glycosylation, acetylation or proteomic cleavage may regulate the protein functions contributing to diverse human proteome (58). These proteins are differentially secreted in response to changes in the cellular microenvironment such as proteins secreted by tumours are differentially expressed compared to normal tissue and further could provide greater insights into cellular physiology and molecular biology, establishing their role as a biomarker (59). To be used as a potential biomarker, individual proteins, as well as the panel of different proteins, show sensitivity and specificity for their application in diagnostics and/or prognostics by different samples, mainly saliva and serum. For instance, actin, myosin (60), resistin *(RETN)* (61), angiogenic factors and matrix-metalloproteinases (62), transferrin (63) and many more proteins were found to be the potential salivary biomarkers for early diagnosis of OSCC. Besides the individual proteins, panels of proteins, such as four proteins panel; matrix metalloproteinase 1 *(MMP1),* kininogen 1 *(KNG1),* annexin A2 *(ANXA2),* and heat shock protein family A *(Hsp70)* Member 5 *(HSPA5)* (64), another panel of three proteins such as *interleukin 1 beta, 6 and 8* (65), and *AHSG, KRT6C* and *AZGP1* (66), a panel of five proteins i.e., *M2BP, MRP14, CD59,* catalase*,* and profilin (67) have also been explored in saliva of patients for their diagnostic and prognostic potential in OSCC.

Another relevant biological fluid widely explored for proteomics biomarker identification is the serum of OSCC patients. The serum helps to detect systemic response, i.e., the change in the proteins that correspond to the disease pathogenesis. By using serum, several molecules have been tested clinically to be used in cancer detection termed “cancer markers” such as carbohydrates antigen (CA)19-9 and CA125 (68). Various cancer biomarkers such as *CA19-9,* carcinoembryonic antigen *(CEA),* squamous cell carcinoma antigen *(SCC Ag)* (69), immunosuppressive acidic protein *(IAP)* and cytokeratin 19 fragment *(Cyfra)* (69, 70) have been explored for the diagnosis of oral cancer. But due to insufficient accuracy, these markers could not be applied to all oral cancer patients for clinical purposes (70).

###### 4.1.1.1.3 Cellular biomarkers

Circulating Tumour Cells (CTCs) and tumour-derived extracellular vesicles (EVs) are among the cellular entities that get activated and released at different time points during carcinogenesis. CTCs represent the entire spectrum of clones present within a tumour. Thus, the transcriptomic and genomic profile of these CTCs provide important information about the heterogeneity of tumour of that patient (38).

Since CTCs are directly disseminated from the primary tumour and preserve its heterogeneity, they have been used as diagnostic biomarkers for micrometastasis and have thus gained a lot of attention from the perspective of personalized biomarkers in a number of malignancies, including OSCC (71). Adding on to this fact, recent work by our group found that CTCs are typically found in OSCC patients even without lymph node metastasis. CTCs presence and their number detected in the patient's blood have been found to be associated with poor prognosis and the recurrence of the disease (72, 73). A higher count of CTCs has been linked to advanced-stage disease, higher risk of metastasis, and decreased overall survival rate (74, 75). They have also been used in real-time monitoring of disease progression, even after resection, to study tumour evolution (76).

EVs contain variety of biological contents such as proteins, lipids, DNAs and RNAs which might play important roles in mediating tumour development and progression in OSCC (77). A study by He et al. showed elevated levels of miR-24-3p in salivary exosomes of OSCC patients (78). Another study on tumour-derived EVs by Ruowei et al. found that EVs regulate inflammatory cytokines such as IL-17A-induced signalling pathways to promote tumour progression in OSCC (79).

##### 4.1.1.2 Classification of biomarkers based on their application in disease analysis

Biomarkers have been utilized in various aspects of disease analysis, such as diagnosis of disease, planning therapy, evaluating the effectiveness of treatment, prognosis, response to treatment and many more. Based on their potential application, biomarkers are further categorized as diagnostic, prognostic, predictive, monitoring, response/ pharmacodynamic, safety, and susceptibility/risk, as discussed in Table 1 below (80).

**Table 1:** Types of biomarkers based on their applications (80, 81).

Types of applications	Purpose	Examples	References
Diagnostic	• To detect or confirm the presence of a disease or a medical condition of interest. • Marks the foundation of precision medicine	Heat shock protein 70 (HSP70): These are network of molecular chaperons with folding catalyst which assist in protein folding. • Promote tumorigenesis by caspase dependent pathway mediated apoptosis suppression. • Expression of the gene significantly differ between control, leukoplakia and OSCC cases and within OSCC between different histological grades and are associated with progression to advanced stage tumor and nodal positivity. TP53 mutation early sign of HNSCC: TP53 tumour suppressor gene regulate cell growth and division, at the time of errors initiate apoptosis or senescence. • Mutation in TP53 cause invasive progression of lesions, a decrease in survival rate and a poor response to cisplatin and fluorouracil based chemoradiation, hence associated with local recurrence after therapy completion	(82, 83)
Prognostic	• A biomarker used to associate likelihood of a clinical event, disease progression, recurrence of a disease/medical condition • In clinical setup, it is routinely used to set trial entry and exclusion criteria for the stratification of high-risk population	NCBP2 and TFRC: High level of these proteins involved in regulating the proliferation, migration and invasion during the course of OSCC	(84)
Predictive	• A change in the biomarkers meant to anticipate the response of patients to a treatment or therapy	Ki-67: Nuclear protein, present in highly proliferating cells but disappear in resting phase of a cell. • Its level is predictive of relapse free and overall survival	(85)
Monitoring	• To assess the status of a disease or medical condition, through the course of the illness. • Change in the value helps predict status of the disease	Desmoglein-3: Preferentially maintains structural integrity in the oral epithelium. Keratin 13: Involved in regulating the differentiation of cells and play major role in carcinogenesis	(86, 87)
Response/pharmacodynamics	• A biomarker whose level changes in response to exposure to a medicine/therapy or an environment agent. • Highly useful in clinics and therapeutic development	Filamin-A(FLNA): Plays a major role in organization of extracellular network that assist in exchange of signals, control DNA double strand break response, cell-ECM interactions, cell signalling. • High expression of FLNA in the buccal squamous cell carcinoma (BSCC) patient tissue associated with poor survival -regarded as a novel biomarker for the diagnosis and outcome prediction of OSCC	(88)
Susceptibility/risk	• A biomarker which can be used to predict the changes of disease development in an apparently healthy individual. • Marks the basis of epidemiological studies on risk prediction	Fibrinogen gamma chain: Major function in homeostasis, significantly higher expression in OSCC compared to healthy controls. α-Defensins 1–3: Major constituent of azurophilic granules of neutrophils, not expressed in normal mucosa, their levels rise in physiological states to exhibit innate immune defenses against infectious diseases including epithelial cancers. • Acts as an important predictor linking inflammation, angiogenesis and cancer	(89, 90)

**Figure 1:**
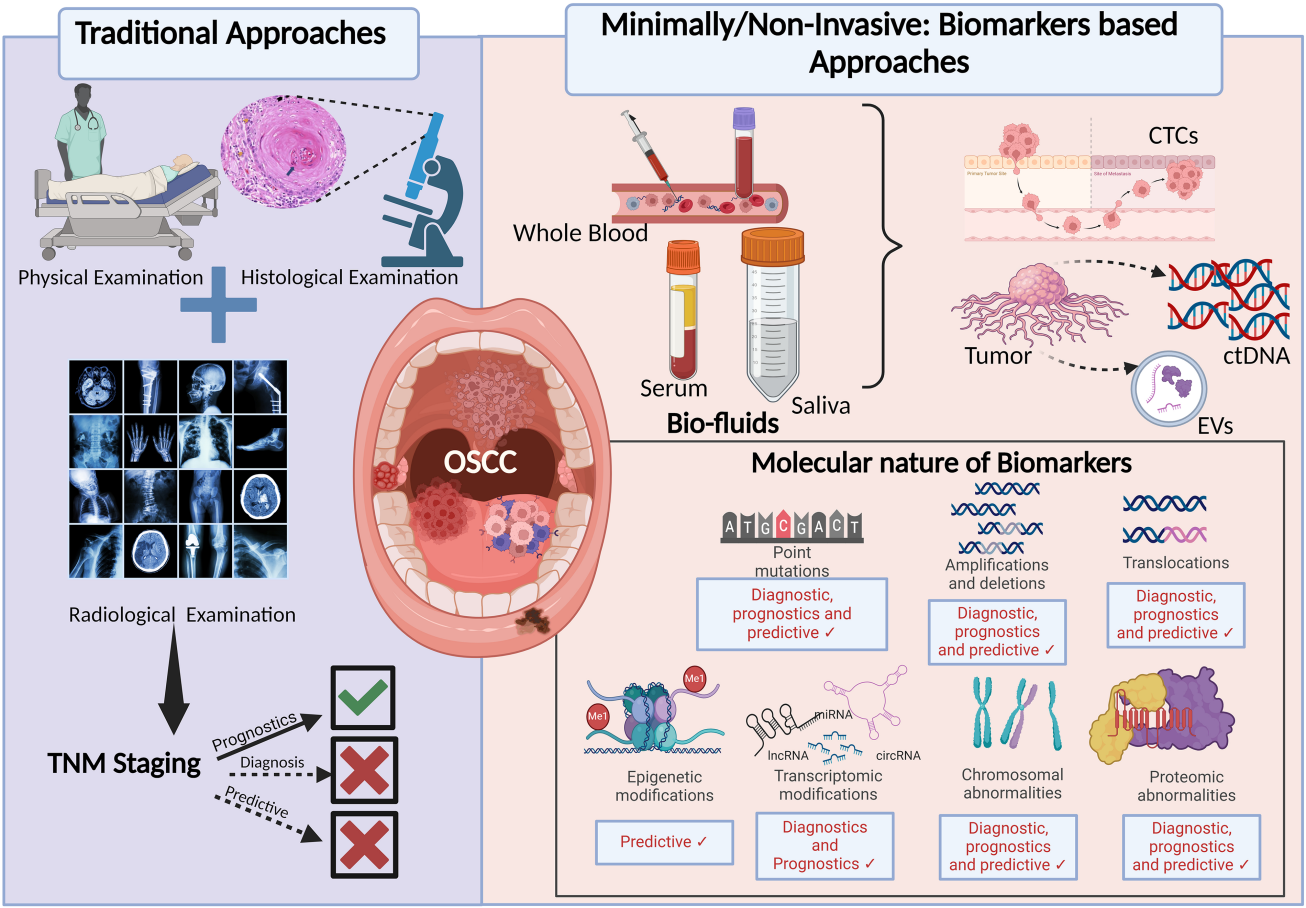
Approaches in the diagnosis and prognosis of OSCC: the figure depicting the advancements from traditional and generalised to recent minimally/non-invasive and personalised approach for the early diagnosis and better prognosis of OSCC. TNM, tumour, node, metastasis; CTCs, circulating tumour cells; ctDNA, circulating tumour DNA; EVs, extracellular vesicles.

Different approaches in the management of OSCC and their applications in patient care are discussed in Figure 1.

## 5 Discussion

OSCC is a heterogeneous group of disorders characterized by neoplastic growth originating from the squamous epithelial lining of the oral mucosa. The prevalence of the disease is ever-rising, and a similar trend is seen in the case of disease-associated mortality. Hence, there is an urgent need to understand the existing challenges and limitations associated with the available management modalities and what modifications need to be incorporated to improve the outcome. With advancements in technology and a better understanding of the disease, more and more management opportunities focused on the molecular characteristics of cancer cells and the effect of the treatment modality on the patient's disease outcome have become the focus of therapeutics. For proper management of OSCC and enhancing the quality of life of the patient, timely diagnosis and treatment with minimal side effects should be the focus. Liquid biopsy-based biomarkers such as cfDNA, non-coding RNAs, proteins and CTCs in the blood, saliva, buccal swabs and other body fluids of the patients are widely being studied for potential use in OSCC. Estimation of the levels of these biomolecules at an early stage of a disease can help in timely diagnosis and further appropriate management of the disease. At times, the assessment of these biomarkers at regular intervals during the course of the disease management can aid in planning treatment, effectively evaluating the disease, and designing personalized therapy.

On one hand, these biomarkers help in the longitudinal assessment of the disease progression and therapeutics design, but on the other hand, their discovery is also associated with challenges. Most of the biomolecules which have been explored for their biomarker potential are still confined to the research stage and have not been expanded to the clinics due to a lack of sensitivity and specificity as well as technical hurdles such as their identification, standardization, and further validation for clinical utility. Moreover, some of these potential markers showing promising results in the early discovery phase failed to reproduce similar successful results during large-scale diagnostic trials.

To attain success in identifying successful biomarkers, including the non-invasive ones, there is a need for the involvement of a multidisciplinary approach comprising experts from clinical oncology, radiology, pathology, and molecular biology specialized in their fields for a collaborative decision-making process, who can harness the available gold standard methods along with these valuable biomarkers and transform the current landscape of OSCC with the best possible solution to the patient.

To summarize, in the era of the ever-growing prevalence of OSCC, prospective use of these biomarkers in the clinical setting will enable early diagnosis and prognosis to enhance the efficacy of cancer management, given that they are highly sensitive, specific and properly validated in large cohorts of patients.
